# Ultrafast Fiber Lasers with Low-Dimensional Saturable Absorbers: Status and Prospects

**DOI:** 10.3390/s21113676

**Published:** 2021-05-25

**Authors:** Pulak Chandra Debnath, Dong-Il Yeom

**Affiliations:** 1 Department of Energy Systems Research, Ajou University, 206 Worldcup-ro, Yeongtong-gu, Suwon 16499, Korea; pcd2k1@ajou.ac.kr; 2 Department of Physics, Ajou University, 206 Worldcup-ro, Yeongtong-gu, Suwon 16499, Korea

**Keywords:** ultrafast fiber laser, saturable absorber, low-dimensional materials, optically/electrically controlled fiber lasers

## Abstract

Wide-spectral saturable absorption (SA) in low-dimensional (LD) nanomaterials such as zero-, one-, and two-dimensional materials has been proven experimentally with outstanding results, including low saturation intensity, deep modulation depth, and fast carrier recovery time. LD nanomaterials can therefore be used as SAs for mode-locking or Q-switching to generate ultrafast fiber laser pulses with a high repetition rate and short duration in the visible, near-infrared, and mid-infrared wavelength regions. Here, we review the recent development of emerging LD nanomaterials as SAs for ultrafast mode-locked fiber laser applications in different dispersion regimes such as anomalous and normal dispersion regimes of the laser cavity operating in the near-infrared region, especially at ~1550 nm. The preparation methods, nonlinear optical properties of LD SAs, and various integration schemes for incorporating LD SAs into fiber laser systems are introduced. In addition to these, externally (electrically or optically) controlled pulsed fiber laser behavior and other characteristics of various LD SAs are summarized. Finally, the perspectives and challenges facing LD SA-based mode-locked ultrafast fiber lasers are highlighted.

## 1. Introduction

Ultrafast lasers have been proven as one of the most effective tools for a wide variety of applications in ultra-precision manufacturing, strong-field physics, nonlinear optics, medical diagnosis, astronomical detection, precision measurement, and fundamental scientific research because of their extremely narrow (femtosecond scale) pulse duration and large peak-power [1,2,3,4,5,6]. Among ultrafast lasers, the passively mode-locked ultrafast fiber laser (MLFL) based on a saturable absorber (SA) has also emerged as one of the most powerful strategies to develop ultrashort pulses (<100 fs) because of their benefits of high beam quality, low cost, efficient structure, alignment-free compact design, and excellent compatibility [6,7,8,9]. Passive mode-locking is a technique that creates a preferred environment for pulsed operation of a laser, effectively employing nonlinear polarization rotation (NPR), nonlinear amplifying loop mirror (NALM), and SA techniques [10,11,12,13]. In particular, the advancement of SA design is principally based on the evolution of materials having SA behaviors. In the recent past, the most extensively used form of SAs includes the semiconducting saturable absorber mirror (SESAM), a semiconducting quantum well structure prepared by a deposition method named molecular beam epitaxy (MBE) [14,15,16,17,18,19]. Modulation depth, SA coefficient, saturation fluence, and recovery time are some of the distinguishing characteristics of a SESAM, determined by adjusting the SESAM’s structure. The high stability of the SESAM turns it into one of the significant choices as SAs. However, several detrimental features, including prolonged recovery time (∼pico-second level), ultra-narrow working wavelength range, low damage threshold, complicated fabrication process, and high cost of SESAM, have guided the scientific community to find new SA materials which can replace SESAM quantum well. The first condition as alternative SA material is to exhibit a nonlinear absorption behavior such that the optical transmittance efficiently increases as the input laser power increases. Other requirements include a high damage threshold, wide operating range, fast recovery time, low cost, and reduced mode-locking threshold, which are vital for the additional advancement of mode-locked ultrafast fiber lasers.

Due to the optical nonlinearity of low-dimensional (LD) materials-based SAs, they are able to modulate and control the circulating light wave periodically in the laser cavity, which results in many longitudinal modes to phase oscillation through ultrafast carrier excitation and the recombination process, thus generating regular ultrashort pulse trains in the time scale. Pauli-blocking plays a critical role in an SA, which reduces the light absorption in the SA instantaneously if a large number of electrons are excited from the lower energy level of the SA to the upper energy level by the incidence of larger light fluence [6,20,21,22]. So far, the emergence of low-dimensional (LD) SA materials, including two-dimensional (2D), one dimensional(1D), and zero-dimensional (0D) materials such as graphene, carbon nanotubes (CNTs), quantum dots, respectively with various advantages over SESAM, provides a new prospect for the development of pulsed fiber lasers because of their distinct structure and physical properties [20,21,22,23,24,25,26,27,28,29,30,31,32,33,34,35,36]. They exhibit divergent physical behaviors varying from semiconductor to insulator and metal to semimetal [37,38,39,40,41,42,43,44,45]. 2D materials, which are mostly studied and investigated among three types of LD materials, have wide-ranging applications in optics, involving ultrafast fiber lasers as well as modulation, generation, propagation, and detection of light [46,47,48,49,50,51,52,53], have been employed to develop integrated photonic circuits.

**Figure 1:**
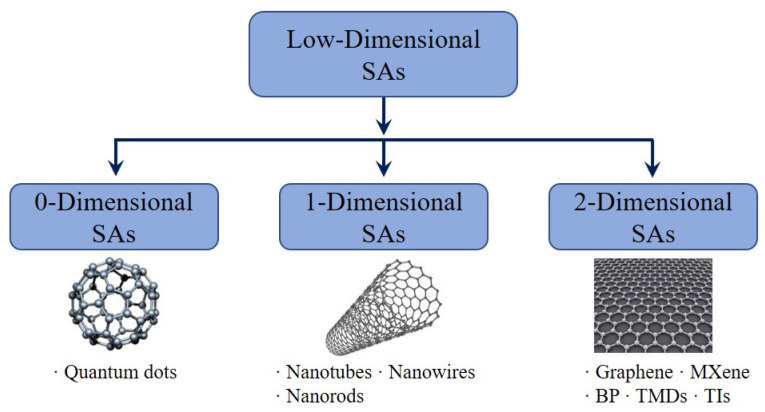
Low-dimensional SAs classification featured as 0D, 1D and 2D structure varieties. [Images are publicly available online].

2D material belongs to the atomic layer material, which can be a monolayer or multiple-layer thick. 2D materials have solid covalent bonding in a layer and weak interlayer van der Waals force. The electrons’ dynamic is limited in the 2D structure if there is no interference of interlayer interaction, which provides 2D materials with numerous novel optical and electrical characteristics [53,54,55]. Graphene is the earliest monoatomic 2D layer material revealed, with remarkable optical, electrical, mechanical, and thermal behaviors [40,41,42,43,44,45]. Following 2D graphene, black phosphorus (BP), transition metal dichalcogenides (TMDs), topological insulators (TIs), perovskite and MXene, and other divergent 2D materials were investigated [8,9,56,57,58]. Moreover, the development of 1D materials such as carbon nanotubes (CNTs) and 0D materials such as quantum dots (QDs) has also made numerous, excellent accomplishments in the development of mode-locked pulsed ultrafast fiber lasers (Figure 1) [22,23,24,28,59,60,61,62,63,64]. Since the CNT and graphene were firstly suggested in 2004 and 2009 as an optical SA for mode-locked ultrafast fiber lasers, respectively [21,22], many other LD materials other than graphene, comprising TMDs, TIs, black phosphorus, bismuthine, MXenes, and metal-organic frameworks, quantum dots have been consecutively investigated, implying the significant growth of new SAs based on LD for ultrafast fiber lasers [8,35,58,65]. In addition to these, externally tunable in-line nonlinear LD SAs, where the fiber laser operation can be tuned from continuous wave, through Q-switched to passively mode-locked regime employing electrical gating or external optical bias, have also been demonstrated using various LD SAs [25,26,27,66,67,68].

Here, in this review, we study the basic characteristics, renowned synthesis methods of the most widely studied LD materials, and fabrication methods for SA devices comprising various coupling techniques to incorporate LD SAs into fiber systems in brief. Afterward, we mainly focus on the advancement in mode-locked (ML) ultrafast fiber lasers based on various LD SAs (especially 2D and 1D) operating in different dispersion regimes at telecommunication wavelengths near ~1550 nm. In addition, we focus on the recent development of externally controlled fiber lasers based on various LD SAs, which operate in several operating regimes such as QS, QS-ML, and ML depending on the external electrical or optical bias to the SA. In the end, we discuss several prospects about the perspectives and potential advancements of ultrafast pulsed fiber lasers based on LD SA materials.

## 2. Properties/Characteristics of LD Materials

### 2.1. 2D Materials

**Figure 2:**
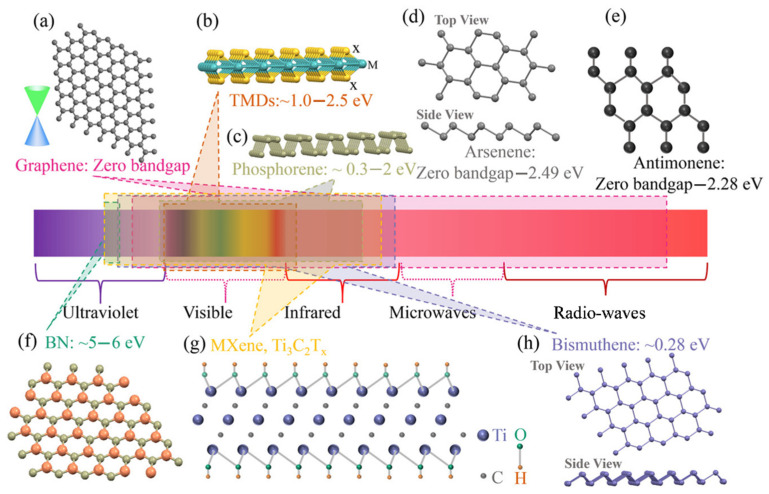
The atomic structure and spectral region of 2D materials. The atomic structure and spectral region of 2D materials such as graphene (**a**), TMDs (**b**), phosphorene (**c**), arsenene (**d**), antimonene (**e**), bismuthine (**f**), MXene (**g**), and BN (**h**). Reproduced with permission [82]. Copyright 2019, Wiley-VCH.

2D materials, in the area of ultrafast nonlinear optics and photonics, are distinguished by their ultrafast recovery, wideband nonlinear saturable absorptions, significant nonlinear refractive indices, and capability as superior mode-lockers for ultrafast fiber lasers [69,70,71,72,73,74,75,76]; what follows is a brief outline of atomic and bandgap structures, and recovery times in 2D materials. Figure 2 illustrates a detailed contrast among the discovered various 2D materials in the family. In this part of the review, fundamental characteristics of related various 2D materials and their incorporation techniques in fiber optic systems are discussed.

#### 2.1.1. Graphene

Graphene, an atomic layered sp^2^-bonded carbon atom arranged in a honeycomb lattice [42,45], is considered as the pioneer of all 2D materials available afterward (Figure 2a), expediting huge prospectives for SA in the research field of ultrafast fiber lasers. Monolayer graphene is estimated to absorb a 2.3% incident light infrared (IR) region owing to its gapless Dirac cone structure [40,44]. Unique characteristics of graphene, such as ultrashort recovery time (<200 fs), low saturable absorption (∼10 MW∕cm^2^ [77]), great relative modulation depth (>60% per layer [21]), and wavelength-independent operation (ranging from the visible to the terahertz), make it special and allow it to perform efficiently as an SA to build wideband mode-locked ultrafast fiber laser pulses.

#### 2.1.2. Transition Metal Dichalcogenides (TMDs)

TMDs with the chemical formula MX_2_, where M refers to a transition metal (e.g., Mo, Nb, Ti, W) and X refers to a chalcogen (e.g., S, Se, or Te), are a group >40 various semiconductors [78,79]. A TMD monolayer is displayed as a layered structure like graphene, where the single transition metal layer is sandwiched in between two chalcogen layers. TMD exhibits an energy bandgap ranging from 1 to 2.5 eV as a group of semiconductors depending on different chemical compositions (Figure 2b). Surprisingly, incident photon energy on TMDs is considerably lower than their normal bandgaps, undergoing significant absorptions due to the carrier excitation within sub-bandgaps formed by the pristine edge states of TMDs [80,81]. In the meantime, TMDs exhibit ultrashort recovery times in the picosecond scale, useful in the field of ultrafast light modulation and ultrafast fiber lasers. For example, MoTe_2_, MoS_2_, WS_2_, and WSe_2_ have been extensively studied and investigated to generate ultrafast fiber lasers in 1.5 and 2 μm wavelength regimes.

#### 2.1.3. Black Phosphorus (BP)

BP is an allotrope of phosphorus and featured as thermodynamically stable at ambient temperature, also called phosphorene for its monolayer case (Figure 2c) [83,84]. BP is formed as a ring structure linked by six phosphorus atoms as with graphene, where each atom is linked to three neighboring phosphorus atoms. Unlike the graphene structure, the structure of BP is puckered, which breaks the symmetry and results in an angle-dependent optical nonlinearity [85]. BP is featured as a direct bandgap semiconductor that is tunable depending on the number of layers (thickness). The bandgap in BP is ranged from 0.35 eV for a bulk case to 2 eV for monolayer BP, which revealed its wideband nonlinear optical response in the MIR regime [86,87], and extensive investigation for ultrafast fiber lasers [24,88]. It has been exhibited that when a BP nanosheet was excited by various wavelength light with photon energies ranging from 1.55 to 0.61 eV, the recovery time depending on the wavelength was varied from 0.36 to 1.36 ps [89]. At room temperature, BP exposed to air in ambient conditions is unstable and degrades its physical properties, requiring high-quality passivation to improve its stability on a long-term basis [90].

#### 2.1.4. Topological Insulators (TIs)

TIs are identified as novel 2D material along with other 2D materials having topological order protected by nontrivial symmetry, which acts as insulators in their inner portion, but gapless conducting states appear on its surfaces [91,92,93]. TIs exhibit effective wideband nonlinear optical responses, like graphene, from the visible to the mid-IR owing to their small indirect bulk bandgap of 0.2–0.3 eV. The phonon-induced carriers’ lifetime of TIs is as short as several picoseconds, revealing them to be used for ultrafast fiber laser and nonlinear optical modulators. Among the various TIs, Bi_2_Te_3_, Bi_2_Se_3_, and Sb_2_Te_3_ are the most-used TIs as SAs.

#### 2.1.5. MXenes

MXenes categorized as a new class of 2D transition metal nitrides, carbonitrides, or carbides, with the composition of M_n1_X_n_T_x_n, where M refers to transition metals (Ti, Sc, Hf, Zr, Nb, V, Cr, Mo, Ta, etc.), X stands for nitride and/or carbide, and T_x_ is surface terminations (O, OH, F, etc.) [94,95]. Few-layer Ti_3_C_2_T_x_ exhibits an indirect energy bandgap lower than 0.2 eV and reduced absorption of around 1%/nm (Figure 2g). 2D MXene materials are usually periodically stacked by means of van der Waals interaction in which no internal termination occurs, a similar feature seen in phosphorene, graphene, and TMDs. In the latest work, it was observed that stacked MXene comprises a set of monolayer MXene in it without any significant disorder [33], implying the possibility of using MXenes as an SA to generate ultrafast fiber lasers, which avoids the deleterious methods of monolayer dispersion. 

#### 2.1.6. Bismuthine

Bismuthine has drawn immense attention in the scientific community, owing to its unique electronic and mechanical characteristics, along with its excellent stability [37,96]. In the latest report, the tunable optical bandgap depending on the layer number varied from nearly 0 to 0.55 eV (Figure 2h) in beta-bismuthine, which reveals bismuthine as an effective wideband nonlinear optical material from the terahertz regions to the near-IR [97]. Comparably shorter recovery time of 2.8 ps in monolayer bismuthine also implies that bismuthine can be a potential candidate as an SA for ultrafast fiber laser applications.

#### 2.1.7. Other 2D Materials

The 2D materials mentioned above display distinctive yet complementary characteristics and, therefore, new opportunities for optical applications in ultrafast fiber lasers [98,99,100]. However, the SA technology required for standard fiber laser operation is always anticipated as perfect as it is theoretically attributed with superior optical properties including ultrashort carrier lifetimes, elevated modulation depths, and damage thresholds. Therefore, it is obvious that exploring novel 2D materials as SA is continuous since it was first implemented. In addition, the controllability of key features in current 2D SAs and engineering in the laser cavity with tunable behavior of SAs also offers more functionalities in the development of controllable ultrafast fiber lasers [25,26,27,66,68,101]. Combining two or more similar or different 2D materials and building van der Waals heterostructures prospects towards developing multi-functional, high efficiency, broadband controllable photonic devices [102,103]. Recently, several ultrafast fiber lasers mode-locked by heterostructure SAs have been reported [104,105].

### 2.2. 1D Materials

#### Carbon Nanotubes (CNTs)

Carbon nanotubes, a unified cylindrical, 1D nanocrystalline graphite material with a high aspect ratio, include a diameter varying from a few to several hundred nanometers and length of up to centimeters. According to the number of tube walls, CNTs can be divided into many types: single-walled CNTs (SWCNTs), double-walled CNTs, and multi-walled CNTs. The chiral properties of carbon tubes lead to different applications of metallic and semiconducting carbon tubes. The carbon tube of the semiconductor type has an obvious bandgap, while the band of the metal type is continuous. CNTs possess multiple excellent properties and advantages that are well fitted with the requirements of a good SA. The measured third-order nonlinear polarizability by pump-probe spectroscopy is 10^−7^–10^−10^ esu (1 esu = 1.11 × 10^−9^ m^2^ V^−2^). The recovery time was measured to be composed of a fast intraband carrier relaxation time of 0.3–1.2 ps and a slow recombination process of 5–20 ps [106]. Moreover, the superior thermal conductivities as high as 5000 W m^−1^ guarantee intrinsic high-power handling. A highly developed growth process significantly lessens the price of raw materials as well as the research cost. More notably, the expansion of CNT SAs over the past 15 years simplified the direction of all-fiber integration configuration, and extensive investigations have validated its operation in a broadband range, which is an underlying disadvantage of industrial SESAMs. Therefore, CNT SAs are a trustworthy candidate to perform as a promising replacement to SESAMs in the future.

## 3. Synthesis of LD SA and Device Fabrication 

### 3.1. Synthesis Techniques

In the past decade, several synthesis techniques have been successfully established to prepare the LD materials introduced in the previous section. The most common synthesis methods for LD materials are categorized into two types: top-down and bottom-up methods. To briefly review, a few standard methods from these types are discussed. The top-down exfoliation methods comprise liquid-phase exfoliation (LPE), mechanical exfoliation (ME), laser thinning, and chemical exfoliation, where single-layer or multiple-layer 2D nanosheets are separated from bulk materials by violating the van der Waals force between layers [9]. Bottom-up methods include pulsed laser deposition (PLD) and chemical vapor deposition (CVD), where high-quality 2D materials in atomic layer scale are effectively synthesized by explicitly adjusting the chemical reactions among solid precursors. Most common and widely applied synthesis and preparation methods for 2D SAs to realize mode-locked fiber lasers will be discussed in brief.

#### 3.1.1. Mechanical Exfoliation (ME)

The ME technique is commonly used in the manufacturing of atomically and few layers thick sheets of 2D layered inorganic materials [44,107,108,109,110]. Researchers can acquire high-quality 2D mono- and few-layer materials by resolving the van der Waals force and splitting layers away from bulk materials. This method was first used in the discovery of 2D graphene from graphite flakes in 2004 by Geim and Novoselov [44] owing to its flexibility and potential to manufacture few-layer materials with outstanding qualities. Compared to the bulk materials, single-layered or multi-layered 2D materials are highly comprehensive and have negligible defects, making them ideal for basic scientific study. Although this method is convenient, fast, and cost-effective, it does have some drawbacks. Since large-area single-layer 2D materials synthesis using this method is difficult, it is only appropriate for fundamental research in a laboratory. Many studies have demonstrated ME using scotch tape to synthesize other 2D materials. This procedure is often used to obtain monolayer BP. Zenghui Wang et al. employed key strategies specifically designed to expedite the transfer of BP after exfoliation to reduce the material’s exposure to the ambient conditions [111]. 

#### 3.1.2. Liquid Phase Exfoliation (LPE)

Liquid phase exfoliation with a high yield has become a viable alternative to mechanical exfoliation [50,112], where large numbers of dispersed 2D layers are exfoliated from its bulk (layered compound) state in liquids. There are four LPE approaches mostly used to eliminating interlayer forces: (i) oxidation followed by ultrasonication, (ii) ion intercalation, (iii) ion exchange, and (iv) sonication-assisted exfoliation. In the oxidation method, layered 2D crystals with low reductive potential are exfoliated by oxidation and subsequent dispersion and ultrasonication in suitable solvents. In ion intercalation, the interlayer gap is expanded by embedding organic or ionic materials as intercalants, such as *n*-butyllithium or IBr in liquids, which disrupt the interlayer adhesion force between the 2D layers in bulk. In ion-exchange methods, layered compounds contain ions between the layers so as to balance the surface charge on the layers. These ions are exchanged in a liquid environment for other larger ions, leading to substantial swelling in layered compounds, and subsequent agitation results in an exfoliated dispersion. The other method is to create microbubbles and pressures between layers of bulk materials directly using high-intensity ultrasonics. However, these approaches can effectively modify the material’s composition in liquids, and the quality must be enhanced further.

#### 3.1.3. Chemical Vapor Deposition (CVD)

**Figure 3:**
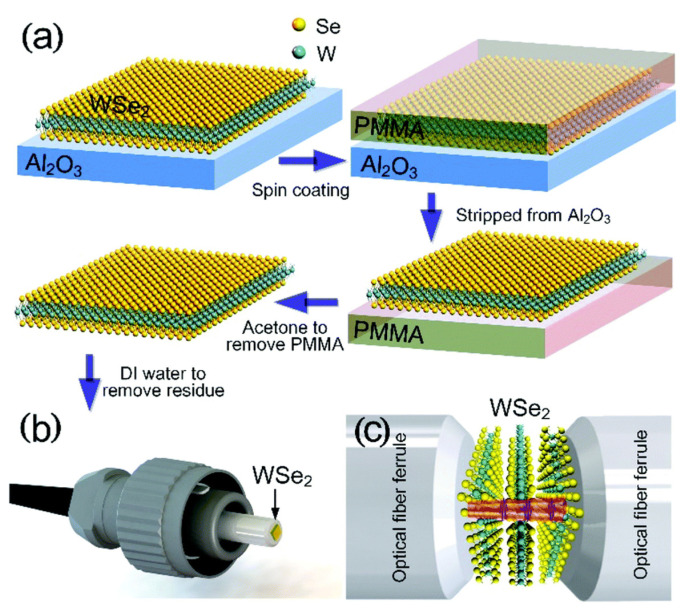
Graphic illustration of the preparation of the WSe_2_-based SA by the CVD method. (**a**) The transfer scheme of WSe_2_ films. (**b**) optical image of WSe_2_ films transferred onto the fiber ferrule end facet. (**c**) schematic presentation of the few-layer WSe_2_ and light interaction. Reproduced with permission [116]. Copyright 2018, RSC.

The uncontrollable scale and random thickness of few-layered 2D materials acquired using the ME or LPE techniques are counterproductive to the efficiency of an SA [113,114]. CVD is an essential bottom-up approach for synthesizing comparatively large area 2D materials with scalability. In 2009, the CVD technique was first employed to synthesize graphene on a copper substrate with a large area (centimeter scale) and high uniformity [115]. Following this synthesis, numerous 2D materials including TIs and TMDs were synthesized employing the CVD method. Few-layer WSe_2_ with a large area and improved quality were synthesized by CVD to be employed as SA in the generation of ultrafast fiber laser, reported by W. Liu et al. as shown in Figure 3 [116]. One of the key features of the CVD technique is its functionality to adjust the number of layers in 2D materials, which elevates the modulation depth of 2D SAs. Nevertheless, this method undergoes deleterious transfer steps from the grown substrate to the fiber system to build the SA device, making this technique complicated to realize cost-effective devices [21,116,117]. To solve these issues, direct synthesis methods have been reported for graphene to build nonlinear optical devices including SA devices [32,101,118,119,120].

### 3.2. LD SAs Integration with Optical Fiber

**Figure 4:**
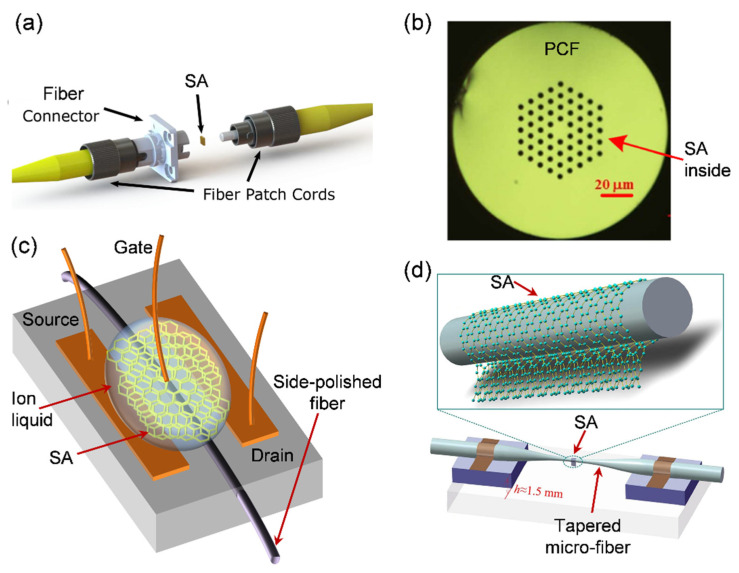
Various schemes for incorporating LD SAs in optical fiber. (**a**) SA sandwiched between two fiber connectors. Reproduced with permission [126]. Copyright 2015, The Optical Society of America. (**b**) LD SA injected inside a hollow photonic crystal fiber (PCFs). Reproduced with permission [125]. Copyright 2013, The Optical Society of America. (**c**) LD SAs transferred onto SPF surface. (**d**) depositing SAs around tapered micro-fiber. Reproduced with permission [127]. Copyright 2016, Springer Nature.

LD materials should be transferred, synthesized, or directly deposited onto optical fibers to build in-line SA devices. Additionally, a significant interaction of LD material with guided light is required for the stable operation of all-fiber mode-locked ultrafast fiber lasers. Usually, these coupling techniques are variously beneficial for different fiber laser schemes. For LD SA-based fiber lasers, the incorporation of SA must be done with the optical fiber or its component. Figure 4 illustrates a variety of mostly utilized fiber coupling methods which are classified into two cases: transmission coupling and evanescent-field coupling.

#### 3.2.1. Direct Coupling

Direct coupling is the most widespread method to realize SA devices, where SA is sandwiched directly between two fiber end facets, as illustrated in Figure 4a. Various LD materials including mechanically exfoliated graphene, CVD-grown graphene or TMDs, 1D CNT, and MBE-grown TIs have been incorporated in fiber end facets using this method in the previous research [121]. Direct coupling facilitates the stable interaction between the guided laser signal and the SA, resulting in reliable mode-locking operation. Nevertheless, if the incident laser power is relatively high, the direct interaction scheme often leads to significant damage to the SA. Thus, a direct coupling scheme with sandwiched SA materials is generally suitable for mode-locked ultrafast fiber lasers operating at low average power.

#### 3.2.2. Evanescent-Field Coupling

To reduce the damage threshold of fiber SAs, a widely used alternative technique called evanescent-field coupling was introduced to incorporate LD SAs onto a tapered fiber and a D-shaped fiber (also called side-polished fiber, SPF) as shown in Figure 4c,d. In 2007, Yamashita et al. for the first time introduced evanescent field coupling of CNTs on SPF and tapered fiber [122,123]. The evanescent field of guided light through the fiber in this scheme interacts with LD SA on the fiber surface. As shown in Figure 4b, SA materials can be filled into a hollow photonic crystal fiber (PCF) and a hollow core fiber (HCF), which are linked to the fiber laser cavity [124,125]. To achieve this, LD SA materials are initially dispersed in a solvent followed by the subsequent filling of SA dispersion into the hollow PCF and HCF. Then the SA-filled PCF or HCF is dried and connected to the fiber laser ring cavity [125]. However, the core size of PCFs being in the μm range, it is challenging to dry the solvent completely inside the core, resulting in elevated insertion losses, hence the unstable performance of ultrafast fiber lasers.

After incorporation of LD SA in the fiber system, the SA module must be examined to ensure the successful incorporation was occurred or not. The most common technique to characterize the SA module is nonlinear transmission to analyze the modulation depth, saturation fluence, and non-saturable loss of the SAs in a fiber setup [20,21,25].

## 4. Ultrafast Fiber Laser Based on LD SA

**Figure 5:**
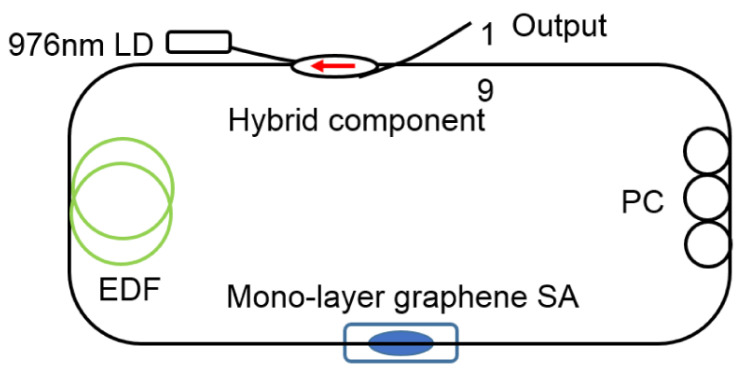
Schematic illustration Er-doped fiber laser ring-cavity comprising a single layer graphene SA on the SPF. LD: laser diode; EDF: erbium-doped fiber; PC: polarization controller; hybrid component: an integrated wavelength-division multiplexer and isolator. Reproduced with permission [128]. Copyright 2015, The Optical Society of America.

In the field of SA-based fiber lasers, mode-locking and Q-switching are two key techniques to generate ultrashort pulses. Both the techniques are useful and selective for various applications depending on their different advantages. Q-switched lasers are mostly utilized in laser processing and military purposes owing to their high pulse energy. On the other hand, because of the ultrashort pulse duration in the femtosecond scale, mode-locked lasers are employed in the areas of nonlinear imaging, micromachining, and fundamental scientific research. Figure 5 shows a comparatively compact and convenient all-fiber laser ring cavity where the optical isolator, output coupler, and wavelength division multiplexer are combined in a hybrid component to reduce the size of the cavity [128]. Over the last decade, numerous LD SAs have been introduced following the invention of graphene and CNT as SA to build ultrafast fiber lasers using the typical fiber laser cavity mentioned above. A summary of various LD SA-based mode-locked Erbium-doped ultrafast fiber lasers’ basic characteristics is provided in Table 1. In terms of the performance of these mode-locked lasers based on graphene, some exciting results have been obtained, including the minimum pulse width and maximum output power of 29 fs [129] and 174 mW [130], respectively. For TIs, the corresponding figures were 128 fs [131] and 75 mW [132], for TMDs they were 67 fs [133] and 57 mW [134], and for BP they were 102 fs [135] and 5.6 mW [114], respectively. Below we introduce the mode-locked ultrafast fiber laser based on several LD SAs and review the advancement of MLFL in terms of controllability in operation, performance, and application.

### 4.1. Ultrafast Fiber Laser Based on Graphene SA

**Figure 6:**
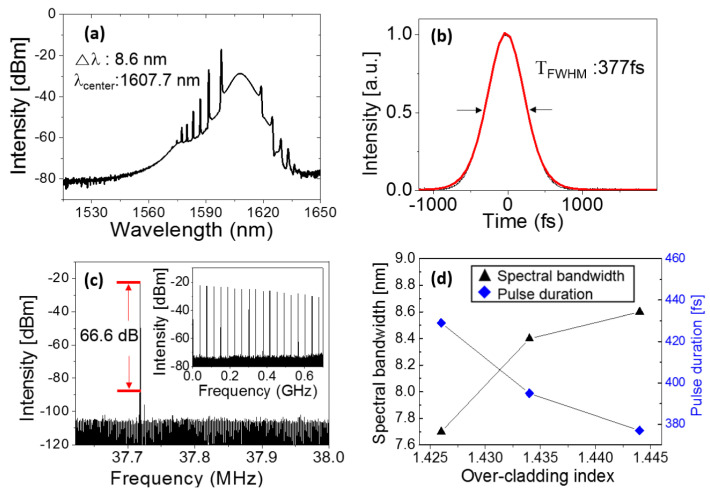
Mode-locked ultrafast fiber laser based on graphene SA (**a**) soliton pulse spectrum and (**b**) autocorrelation trace of the mode-locked pulse. (**c**) RF spectrum of the laser output pulse train (inset: RF spectrum viewed over a wide frequency range) and (**d**) 3-dB bandwidth and pulse width of the implemented laser as functions of the over-cladding index of the SA. Reproduced with permission [128]. Copyright 2015, The Optical Society of America.

After the discovery of graphene, it has been widely studied and investigated in various scientific research fields because of its distinctive nonlinear optical properties [31,54,136]. The features of broadband optical absorption, ultrafast carrier recovery, gapless band structure, and high damage threshold make graphene one of the leading SAs in the application of fiber laser lasers. First, the convergence of valence band and conduction band at the Dirac point of graphene denotes a gapless semi-metallic band, i.e., zero-bandgap structure, which results in graphene being conducive to wideband absorption. Secondly, the presence of an exclusive quantum tunneling effect in graphene carriers implies fast relaxation time and higher carrier. The relaxation time was investigated through pump-probe experiments in graphene by Bao et al., and the fast relaxation time of 150 fs has been measured in the case of graphene [137]. They exhibited that, to shape ultrashort laser pulses in fs scale, the graphene with a fast relaxation time is more effective. Finally, graphene has a melting point of up to 4510 K [138]. In the experiment, a high damage threshold with graphene SA indicates an appropriate application of graphene SA in high-power lasers. To date, numerous studies and investigations with graphene in ultrafast fiber lasers have been performed. Bao et al. designed and created graphene SA devices for the first time. They investigated graphene SAs as nonlinear optical materials which were employed to generate stable mode-locked ultrafast Erbium-doped fiber laser (EDFL) with 3 dB bandwidth of 5 nm operating at 1565 nm. Following this work, graphene SA-based MLFL has been a hot topic to investigate the various schemes and techniques for controllable MLFL based on graphene SA. In addition, Park et el. utilized the evanescent field coupling of monolayer graphene to produce graphene SA on SPF [128]. Through numerical study, they showed that a strong optical absorption of more than 90% can be achieved without significant scattering loss in a monolayer-graphene saturable absorber by employing an index-matched over-cladding structure on the graphene/SPF. By tuning the index of over-cladding, they effectively controlled and tuned the light coupling into the laser cavity, which results in significant control of the MLFL characteristics. Figure 6 shows the optical spectrum of the laser output, where the measured average output power was 5.41 mW at an applied pump power of 120 mW. The typical spectral shape of an optical soliton was observed, where the measured spectral bandwidth was 8.6 nm at 1607.7 nm. Assuming a soliton pulse, the pulse duration measured by an intensity autocorrelator was 377 fs, as shown in Figure 6b, the fundamental repetition rate was measured to be 37.72 MHz (Figure 6c). The pulse width of the mode-locked laser reduced with the over-cladding index increased, where the measured pulse widths were 429, 395, and 377 fs for the over-cladding indices of 1.426, 1.434, and 1.444, respectively (Figure 6d).

**Table 1:** Summary of the characteristics of various LD SA-based ultrafast fiber lasers operating at ~1.55 μm.

SA	Center Wavelength (nm)	3 dB Bandwidth (nm)	Pulse Duration (ps)	Repetition Rate (MHz)	Output Power (mW)	Pulse Energy (nJ)	Ref.
Graphene (2D)	1559.12	6.16	0.432	25.67	-	0.09	[139]
	1566	4.92	0.88	6.22	-	-	[140]
	1555	6	0.59	45.88	0.91	-	[141]
	1545	48	0.088	21.15	1.5	0.071	[142]
	1553	3	1	8	1	0.125	[143]
	(CS)1565 (DS)1559	7 10.4	13.8	25.8 16.99	0.7 174	10.2	[130]
	1607.7	7.7–8.6	0.37~0.429	37.72	5.41	-	[128]
CNT (1D)	1547.5	0.3	22.73	10.61	11.21	1.057	[144]
	1560.1	4.3	0.763	62.2	0.445	0.007	[145]
	1555.1	3.9	0.85	10.89	3.19	0.29	[146]
	1564.5	5	0.57	18.3	0.316	0.017 pJ	[147]
	1563	12.1	12.7	9.8	335	34	[60]
	1560	4.83	0.602	11.25	8.58	0.763	[64]
	1560	4.33	22.2	0.51	4	0.18	[61]
	1560	42	0.093	38.117	11.2	0.3	[148]
BP (2D)	1569.24	9.35	0.280	60.5	-	-	[149]
	1566.5	3.39	0.94	4.96	5.6	-	[114]
	1561.1	3.25	0.8	5.86	0.3	0.051	[150]
	1555	40	0.102	23.9	1.7	0.071	[135]
Bi_2_Se_3_ (TI)	1557.5	4.3	0.66	12.5	1.8	0.144	[30]
	1558.3 1557.4 1559.4	0.9	3.01 3.42 2.02	5.1 (HML)388 (HML)239	-	-	[151]
	1600	7.9	0.36	35.45	0.86	-	[152]
	1554.56 1559	7.91 26	0.908 7.564	(CS)20.27 (DS)7.04	5.5 75	0.27 0.27	[132]
	1562.4	4.28	0.630	23.3	-	0.0156	[153]
	1557.908	0.342	7.78 ns	1.71	82.6	48.3	[154]
Bi_2_Te_3_ (TI)	1570	5.88~6.66	0.403	28.5	-	-	[155]
	(DS)1560	5.6	2.7 ns	1.7	32.9	19.3	[156]
	(CS)1558.5 (HML)1558.5	0.95 1.08	1.22 2.49	4.88 2.04 GHz	5 5.02	1.02 -	[157]
	1547	4.63	0.6	15.11	0.8	0.0529	[107]
	1558.459	1.696	3.22 ns	1.704	40.37	23.9	[158]
	1560.8	9.15	0.286	18.55	0.5	0.027	[159]
MoS_2_ (TMD)	1570.1	2.7	1.36	5.924	3.5	0.59	[29]
	1571.8	3.5	0.83	11.93	5.85	0.49	[160]
	1574.6	9.5	0.79	29.5	4.13	0.14	[161]
	1568 1568	23.2 12.38	4.98 0.637	(DS)26.02 (CS)33.48	-	-	[162]
	1556.86	2.47	-	6.77	0.065	0.01	[163]
	1560	20.5	0.2	14.53	1	0.069	[113]
	1569.5	4	0.71	12.09	-	-	[164]
	1556.3	6.1	0.935	463	5.9	-	[165]
	(CS) 1530.4	2.1	1.21	8.968	-	-	[166]
	(BS)	(period) 2	1.2	8.968	-	-	
MoSe_2_ (TMD)	(CS)1557.3 (HML)1557.3	5.4 5.1	0.798 0.751	15.38 3.27 GHz	- 0.23~22.8	- 14.6~6.7 pJ	[167]
	1560	7.8	0.580	8.8	-	0.0913	[168]
	1552	12.72	0.207	64.56	-	-	[169]
	1558.35	2.9	1	16.27	-	-	[170]
MoTe_2_ (TMD)	1561	24.9	0.1119	96.323	23.4	-	[171]
	1532.5	1.5	2.57	6.95	1.7	-	[172]
	1559.57	11.76	0.229	26.601	57	2.14	[134]
WS_2_ (TMD)	1565	8.23	0.332	31.11	0.43	-	[173]
	1566	5.6	0.457	21.07	0.32	-	
	1540	114	0.067	135	-	-	[133]
	1572	5.2	0.595	25.25	-	-	[174]
	(DS)1565.5	14.5	21.1	8.05	1.8	0.22	[175]
	1558.5	-	0.675	19.58	0.625	-	[176]
	1563.8	5.19	0.524	19.57	2.64	0.134	[177]
WSe_2_ (TMD)	1556.42	6.06	0.477	14.02	-	-	[117]
WTe_2_ (TMD)	1556.2	4.14	0.77	13.98	-	-	[178]
MXene	1550	42.54	0.104	20.03	-	0.065	[179]
	1567.3	3.1	0.946	8.24	-	-	[56]
	1557	5	0.66	15.4	0.05	-	[33]
	1555.01	22.2	0.159	7.28	3	0.41	[180]
	1565.4	3.4	5.3	8.25	-	-	[181]
BP QD (0D)	1561.7	3	0.882	5.47	-	-	[24]
	1560.3	2.2	1.2	5.62	2.23	0.45	[182]

The corresponding spectral bandwidth changed from 7.7 nm to 8.4 nm to 8.6 nm, indicating that the SA possesses different pulsating abilities depending on the over-cladding index, which affects the pulse formation for a given laser cavity. Consequently, this may allow one to control the soliton pulse bandwidth and pulse width of the generated ultrafast laser through appropriate selection of the over-cladding material with various refractive indexes.

Although ultrafast fiber lasers based on graphene SA have been developed extensively, numerous deficiencies with graphene SA have progressively been revealed through growing investigation. Firstly, the absorption efficiency of 2.3% per layer results in reduced modulation depth which limits its advanced application in ultrafast fiber lasers. Even if a higher number of graphene layers elevate the modulation depth, the unsaturated loss, in this case, declines the performance of the fiber laser. Secondly, the tunable operating wavelength plays a critical role in the saturation threshold of graphene SA. A shorter operating wavelength exhibits a higher saturation threshold in graphene SA. This implies graphene as comparatively proper SA for fiber laser operating in the mid-infrared range but marginally worse performance while working in shorter wavelengths.

### 4.2. Ultrafast Fiber Laser Based on CNT SA

**Figure 7:**
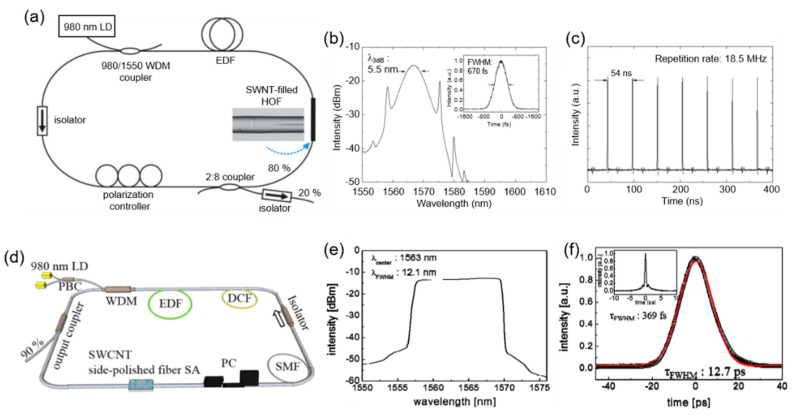
1D SWCNT SA-based mode-locked fiber laser: (**a**–**c**) Conventional soliton: (**a**) Schematic of the fs fiber laser using the SWCNT-filled HOF. The inset figure shows the spliced image between the normal SMF and HOF, where adiabatic mode transition occurs. (**b**) Measured optical spectrum and pulse duration (inset) of the mode-locked fiber laser. (**a**–**c**) Reproduced with permission [188]. Copyright 2009, The Optical Society of America. (**c**) The output pulse train of the laser shows a repetition rate of 18.5 MHz. (**d**–**f**) Dissipative soliton: (**d**) Configuration of the fiber ring laser including the SWCNT-SA and the DCF. (**e**) The optical spectrum of the mode-locked laser at net cavity dispersion of 0.087 ps^2^ and (**f**) Measured pulsed duration fitted with Gaussian pulse. The inset shows the pulse compressed by additional SMF at extra-cavity. (**d**–**f**) Reproduced with permission [60]. Copyright 2014, The Optical Society of America.

The first verification of CNTs as SA in an ultrafast fiber laser system took place in 2003 by Set et al., and ultrashort pulses of ∼1 ps were demonstrated in 1550 nm [183]. Subsequently, CNT SAs have been rapidly adopted by many research groups. The most popular type of SWNT SA used in fiber lasers concentrates on polymer composite film, which is mechanically or optically sandwiched between fiber connectors. Another approach based on the evanescent field can be a candidate, and SWNTs can be coated on a D-shaped fiber [122], microfiber [184], or injected into micro slots [185,186], photonic crystal fiber [187], and hollow-core optical fiber (HOF) [188]. Choi et al. demonstrated the nonlinear interaction scheme of SWCNTs with laser light by using the HOF, which offers the advantages of robust, efficient, and long interaction of guided light with SWCNTs with a simple fabrication process (inset of Figure 7a) [188]. The HOF filled with low concentration SWCNT/polymer composite exhibiting broadband absorption is prepared as an in-line SA, resulting in a passively mode-locked fiber laser with the spectral bandwidth; the pulse duration and repetition rate of the laser output are 5.5 nm, 490 fs, and 18.5 MHz, respectively (Figure 7b,c) [188]. In another demonstration, a dissipative soliton fiber laser with high pulse energy (>30 nJ) based on a single-walled carbon nanotube saturable absorber (SWCNT-SA) on SPF was reported by Jeong et al. [60]. 

A laser cavity generates a dissipative soliton mode-locked pulsed laser only when the laser cavity net dispersion is in the normal dispersion regime. A dispersion compensating fiber (DCF) with large normal dispersion at 1550 nm was inserted along with SMF-28e (anomalous dispersion at 1550 nm) in the cavity to realize the normal net cavity dispersion, as shown in Figure 7d. Stable passive mode-locking of a dissipative soliton laser was obtained at a net cavity dispersion of around 0.141 ps^2^. The generated dissipative soliton exhibits spectral bandwidth of 12.1 nm with a flat top spectral shape at the central wavelength of 1563 nm, as shown in Figure 7e. The flat-top behavior of the generated soliton pulse is evidence of dissipative soliton mode-locked pulsed laser. The laser stably delivers linearly chirped pulses with a pulse duration of 12.7 ps (Figure 7f), and the average power of the laser output is measured as 335 mW at an applied pump power of 1.27 W. The corresponding pulse energy is estimated to be 34 nJ at the fundamental repetition rate of 9.80 MHz; this was the highest value reported in all-fiber Er-doped mode-locked laser using an SWCNT-SA.

### 4.3. Ultrafast Fiber Laser Based on Other 2D SAs

**Figure 8:**
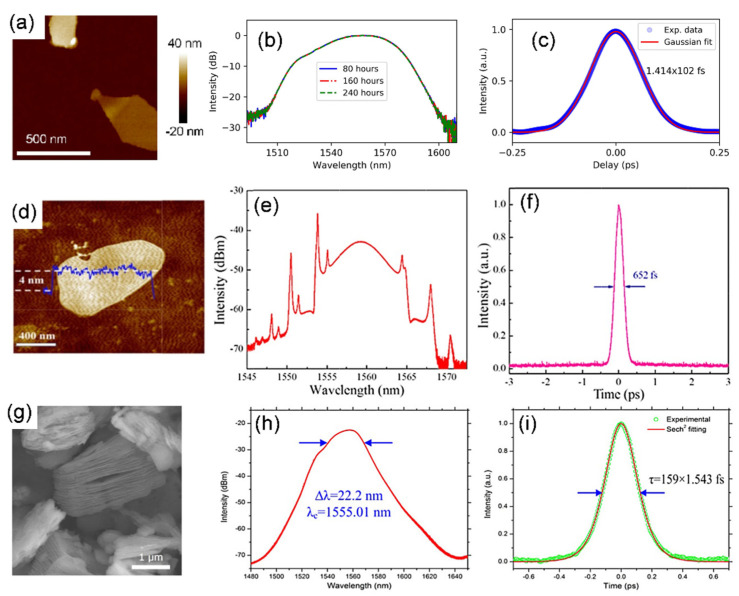
Mode-locked ultrafast fiber laser in the NIR region with various 2D SAs such as BP (**a**–**c**), Bismuthine (**d**–**f**), and Ti_3_C_2_T_x_ MXene (**g**–**i**). (**a**) AFM micrograph of selected exfoliated BP fakes; (**b**) Optical spectrum with a bandwidth of 40 nm acquired after 80 h (blue curve), 160 h (red dot line), and 240 h (green dot line), respectively; (**c**) Autocorrelation trace with a Gaussian fit. (**a**–**c**) Reproduced with permission. Copyright 2018, The Optical Society of America [135]. (**d**) AFM image of few-layer bismuthine. (**e**) mode-locked soliton pulse spectrum with 3 dB bandwidth of 4.64 nm, and (**f**) autocorrelation trace of mode-locked ultrafast laser showing the pulse width of 652 fs. (**d**–**f**) Reproduced with permission. Copyright 2018, Wiley-VCH [191]. (**g**) SEM image of Ti_3_C_2_T_x_, (**h**) mode-locked soliton pulse spectrum with 3 dB bandwidth of 22.2 nm, and (**i**) autocorrelation trace of mode-locked fiber laser showing the pulse width of 159 fs. (**g**–**i**) Reproduced with permission. Copyright 2017, Wiley-VCH [180].

Following an extensive investigation with 2D graphene SA and 1D CNT SA for ultrafast fiber laser, other LD materials-based SAs have also attracted attention to build ultrafast mode-locked fiber laser. In 2014, black phosphorus started to achieve prevalent curiosity due to its unique electro-optical properties [84]. The interesting feature of BP is its band structure which can vary depending on the thickness or number of BP layers. The bandgap of BP reduces with increasing the number of layers or BP thickness because of interaction among the BP layers. The bandgap of bulk BP is 0.3 eV, which estimates that BP is a highly suitable contender for both mid-infrared and near-infrared SAs. One of the unique features of BP is its direct bandgap regardless of the changes in thickness. Compared to graphene, the relaxation time in BP has been found to be faster in the mid-infrared and near-infrared region revealed by Wang et al. using a pump-probe experiment, which suggested the higher ability for pulse narrowing compared to graphene [89]. Consequently, BP, with its direct bandgap feature, provides valuable aspects towards the application of ultrafast nonlinear optics and optoelectronics. Numerous ML ultrafast fiber lasers based on BPs have been successfully investigated and recognized. Chen et al., for the first time, successfully constructed a BP-based SA and engaged it in a fiber laser ring cavity to generate stable ultrafast mode-locked fiber laser operating at 1571.45 nm with a pulse width of 946 fs and 3 dB bandwidth of 2.9 nm [110]. The stability of the laser was confirmed by a signal-to-noise (SNR) ratio of 70 dB. Along with the EDFL operating at 1.55 μm, BP-based SA has also been examined in the 1 μm region achieved by YDFL. Hisyam et al. demonstrated MLFL using BP SA in a YDFL ring cavity, which generates 1 μm mode-locked soliton pulse with 7.54 ps and the highest ever output power of 80 mW at their time of report [189]. The pulse energy was measured to be 5.93 nJ. Pawliszewska et al. obtained 2 μm holmium-doped all-fiber lasers having a pulse duration of 1.3 ps, centered at 2094 nm with a bandwidth of 4.2 nm using BP SA [190]. Significant work on BP SA in the divergent wavelength range of 1–2 μm reveals the broadband absorption characteristics of BP. Jin et al. achieved the shortest pulse duration using BP-based SA at the time of their report [135]. A highly functional inkjet printing technology with high scalability was employed to fabricate the SA based on BP. The exfoliated BP flakes are shown in Figure 8a. Ultrafast fiber laser with this BP SA in an EDFL ring cavity exhibited the pulse width of 102 fs, 3 dB bandwidth of 40 nm at 1555 nm, and as shown in Figure 8a–c. Although the ultrafast fiber laser based on BP SA has been developed extensively with its wideband absorption characteristics and the ability to shorten the pulse width, numerous deficiencies with BP SA have progressively been revealed through growing investigation. Mainly, its instability to environmental factors such as humidity and temperature is detrimental to the fiber laser system. The physical properties of BP are highly sensitive to air. BP increases in its volume unexpectedly if it is exposed to air due to its large affinity for water.

Consequently, the BP surface decayed as time goes by if it was not efficiently passivated for a long time [192]. The optical properties of BP are certainly affected by its environmentally unstable behavior, thus influencing the execution of high-performance fiber lasers with BP SA. Moreover, the BP SA is prone to damage under high power laser due to its unavoidable thermal effects in the air, further limiting its application in elevated power regimes [114].

Inspired by the domination of 2D graphene as an SA for fiber lasers, few-layer 2D bismuthine also has been introduced by Lu et al. as an SA, owing to its direct bandgap at 1550 nm. Bismuthine’s optical bandgap is tunable and controlled by changing the number of layers. By increasing the layer number from one to six layers, it exhibits an optical bandgap varying from 1.028 eV to 0.747 eV [191]. They investigated few-layer bismuthine using various tools including atomic force microscopy (AFM), which confirmed the thickness of prepared few-layer bismuthine SA as 4 nm with a smooth surface as shown in Figure 8d. Generated ultrafast mode-locked fiber laser in the anomalous dispersion regime operating at 1559.18 nm has a spectral bandwidth of 4.64 nm and pulse duration of 652 fs (Figure 8e,f)

MXene, as a recently developed new 2D material, has attracted considerable attention because of its graphene-like but highly tunable and tailorable electronic/optical properties. Researchers found that a typical MXene has efficient SA with negligible lossy nonlinear absorption components in the spectral range 800–1800 nm, which is indicated in a typical MXene, Ti_3_C_2_T_x_, which was deliberately chosen as the investigation object to highlight broadband nonlinear optical response in the near-infrared region [180]. In 2018, Jiang et al. investigated the broadband non-linear photonics of Ti_3_C_2_T_x_ by depositing Ti_3_C_2_T_x_ solution onto a side-polished fiber [180], where stable mode-locking was achieved in both Yb- and Er-doped fiber lasers operating at the wavelength of ~1 μm and ~1.55 μm, respectively. Er-doped fiber laser results are depicted in Figure 8g–i. Highly stable self-started CWML is readily obtained when the pump power is above 60 mW. The ultrashort pulse duration of 159 fs was obtained with the spectral bandwidth of 22.2 nm operating at 1555.05 nm. The output power, pulse energy, and peak power at the pump power of 238 mW are 3 mW, 410 pJ, and 2578.6 W, respectively.

### 4.4. Ultrafast Fiber Laser Based on TMD and TI SAs

**Figure 9:**
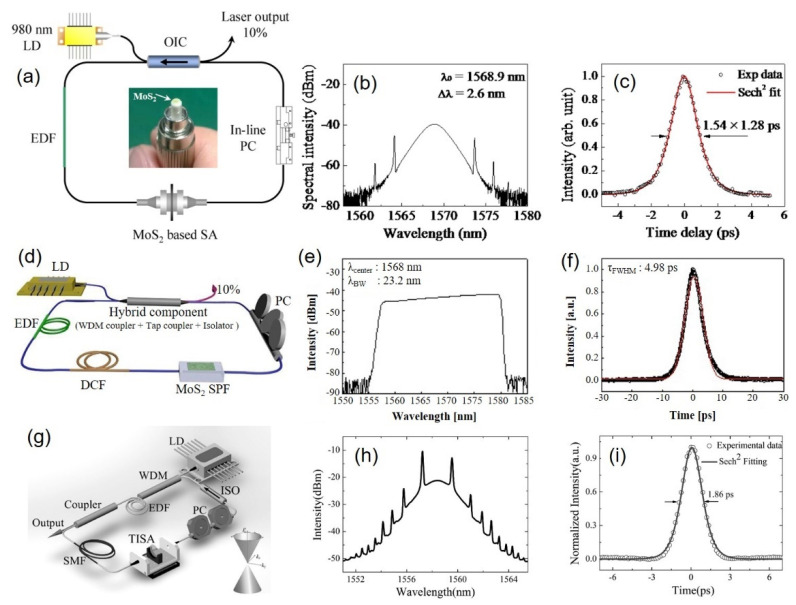
Mode-locked ultrafast fiber laser in the NIR region with various 2D SAs such as one of the TMDs named MoS_2_ SA (**a**–**f**) and one of the TIs named Bi_2_Te_3_ (**g**–**i**). (**a**–**c**) CS with MoS_2_ SA: (**a**) A schematic diagram of the fiber ring laser in anomalous dispersion with MoS_2_ SA sandwiched in between two SMF (inset: Photograph of a fiber connector coated with multilayer MoS_2_); (**b**) Optical spectrum of conventional soliton mode-locked pulse with a bandwidth of 2.6 nm; (**c**) Autocorrelation trace with a Sech^2^ fit showing pulse duration of 1.28 ps. (**a**–**c**) Reproduced with permission. Copyright 2014, The Optical Society [200]. (**d**–**f**) DS with MoS_2_ SA (**d**) A schematic diagram of the fiber ring laser in normal dispersion with MoS_2_ SA deposited on SPF. (**e**) The optical spectrum of the mode-locked laser at net cavity dispersion of +0.095 ps^2^ and (**f**) Measured pulsed duration fitted with Gaussian pulse. (**d**–**f**) Reproduced with permission. Copyright 2014, The Optical Society [162]. (**g**–**i**) CS with Bi_2_Te_3_ SA: (**g**) mode-locked fiber laser cavity comprising Bi_2_Te_3_ SA, (**h**) mode-locked optical spectrum with 3 dB bandwidth of 2.69 nm, and (**i**) autocorrelation trace of mode-locked soliton pulse with the FWHM width of 1.86 ps. (**g**–**i**) Reproduced with permission. Copyright 2012, AIP [201].

TMDs, because of their various types with numerous members in the group, have inhabited the effective prominence as prospective contenders of SAs [162,175,178,193,194,195,196,197]. Among other candidates after graphene, which has been extensively researched in the nonlinear optics field, TMD is discovered to execute well in terms of ultrafast carrier dynamics, switchable bandgap, and higher-order nonlinear optical response. At present, some TMD materials such as MoS_2_, MoTe_2_, MoSe_2_, WSe_2_, and WS_2_ have produced crucial breakthroughs in ultrafast fiber lasers [162,175,178,193,194,195,196,197]. For example, the MoS_2_ bandgap transforms from indirect to direct while the thickness decreases from bulk to monolayer, along with the bandgap rising from 1.8 to 1.29 eV [198]. Moreover, MoS_2_ exhibits a larger third-order nonlinear optical response compared to graphene. In addition to this, MoS_2_ highlights a carrier lifetime of nearly 100 ps and ultrafast intraband relaxation time as short as 30 fs [196]. Table 1 includes the properties of ultrafast fiber lasers with various TMD SAs. Among these TMDs, layered MoS_2_ was first studied. In 2014, the SA behavior of few-layer MoS_2_ was initially observed [194,197,199]. Both the conventional soliton and dissipative soliton mode-locked fiber laser have been reported by individual groups using anomalous dispersion and normal dispersion fiber laser ring cavity, respectively. Xia et al. demonstrated an Er-doped ultrafast fiber laser passively mode-locked by a multilayer MoS_2_ SA prepared by a CVD method and transferred onto the end-face of a fiber connector to build a conventional soliton mode-locked fiber laser pulses operating at 1.57 um wavelength in anomalous dispersion regime as shown in Figure 9a–c [200].

Resultant output soliton pulses showed central wavelength, spectral width, pulse duration, and repetition rate of 1568.9 nm, 2.6 nm, 1.28 ps, and 8.288 MHz, respectively. Khazaeizhad et al., in another work, successfully employed a CVD-grown multilayer MoS_2_ SA in a passively mode-locked Er-doped fiber laser to achieve both soliton and dissipative soliton pulses [162]. Their dissipative soliton pulses characteristics operating at the near infrared region are shown in Figure 9d–f. The normal dispersion cavity was achieved by optimizing the net dispersion of the cavity by adding a segment of dispersion compensating fiber (DCF) in the cavity, as seen in Figure 9d. The stable dissipative soliton pulses with 4.98 ps pulse width at the repetition rate of 26.02 MHz showing a broad spectral width of 23.2 nm. In anomalous dispersion regime, they also obtained soliton pulses with pulse duration of 637 fs at the repetition rate of 33.48 MHz, and with a spectral width of 12.38 nm in an anomalous dispersion cavity. These findings have greatly promoted the development of few-layer MoS_2_ in mode-locked lasers, leading to significant progress in this area.

As new LD graphene-like materials, TIs have been discovered with an energy band structure of symmetry Dirac cone due to their strong spin-orbit interaction, which implies that they can be developed into a new kind of SAs [202,203]. TIs have a nonzero bandgap and a large modulation depth (up to 95%), which are beneficial for improving the performance of mode-locked fiber lasers. Various mode-locked fiber lasers based on TIs, including Bi_2_Se_3_ [204,205], Bi_2_Te_3_ [107,108], and Sb_2_Te_3_ [53], have been developed, most of them are summarized in Table 1 with their mode-locked ultrafast EDFL. TIs were also confirmed as possessing excellent nonlinear optical properties and were used as SAs for demonstrating the ultrafast fiber laser in 2012 [201], as shown in Figure 9g–i. Zhao et al. reported in a first example among all TI that Bi_2_Te_3_ SA exhibited very-high-modulation-depth (up to 95%) saturable absorber and used as a passive mode locker for ultrafast pulse formation at the telecommunication band as shown in Figure 9g–i. In an erbium-doped fiber laser with the help of this SA, self-started mode-locked pulses centered at 1558.4 nm with a pulse width of 1.21 ps could be directly generated out of the laser cavity. These results indicate that, in addition to their established attractive electrical and thermal properties, TIs also have attractive application prospects for an ultrafast fiber laser. 

All these LD SA devices also have been studied to build Q-switched or Q-switched mode-locked fiber laser by changing the cavity conditions such as polarization state, pump power or even by changing the fiber length of the cavity [67,68]. That means there is an additional regime of the fiber laser using these LD SA, including Q-switching and bound-state soliton mode-locking, to achieve high repetition rate fiber lasers in the gigahertz ranges useful for diverse applications. Therefore, the controllability of the fiber laser operating at different regimes using these LD SA would be of great interest due to its compatibility with various fiber-optic systems.

## 5. Externally Controlled Ultrafast Fiber Laser

To date, there are very few investigations reported on the active control of fiber laser using the LD SA, such as graphene and CNT [25,26,27,66,67,68]. These can be done both optically and/or electrically. It is noted that most of these active controls were done for evanescent field interaction cases, as it provides the facilitated device fabrication on the SPF surface along with the high functionality owing to the effective polarization sensitivity of evanescent field wave incident on SPF surfaces. It has been demonstrated that the fermi level of LD SA can be shifted by applying external gate bias to the LD-based capacitor structure, which plays a crucial role in tuning the modulation depth of the SA hence effectively control the fiber laser operating regime [25,67]. Apart from the evanescent field interaction cases, few attempts to simplify the design of actively mode-locked lasers have been made by employing a compact LD SA-based electro-optic modulator which controls the linear optical absorption as well as the modulation depth of the device upon applied external electrical signal [26,27]. These electro-optic modulators have been utilized in fiber laser for active control of pulse generation with tunable repetition rate along with mode-locking and harmonic mode-locking (HML) operation. As for the external optical bias, the nonlinear absorption in the SA can be substantially controlled via cross-absorption modulation (XAM) using evanescent field interaction; thus, the fiber laser operation was optically controlled [68]. A few of the recent works on externally controlled 2D SA for tunable fiber lasers and their characteristics are summarized in Table 2. The following section will briefly review those works covered by both electrically and optically controlled switchable fiber lasers.

### 5.1. Electrically Controlled Gate-Tunable Fiber Lasers

**Figure 10:**
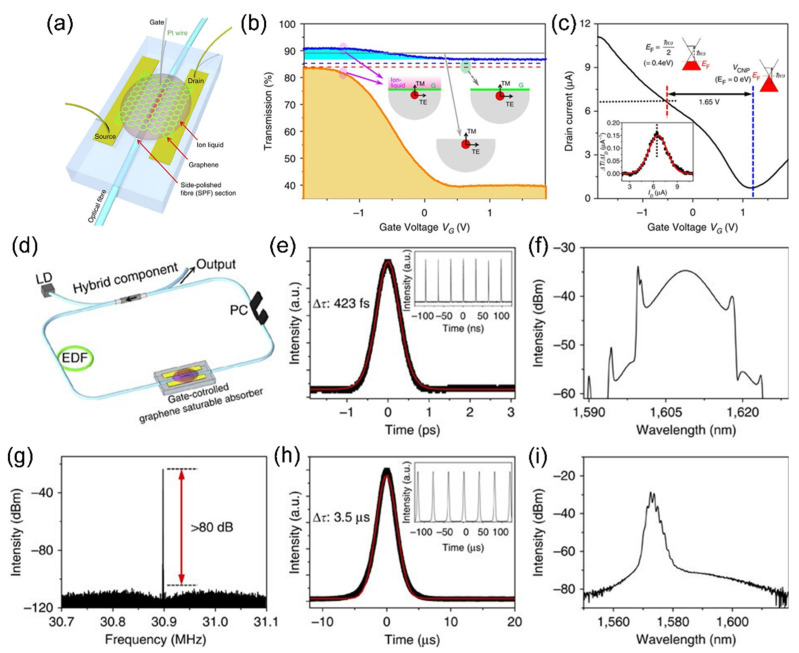
Electrically controlled fiber laser using an all-fiber graphene device and gate-variable properties of fiber laser operation. (**a**) Schematic diagram of gate-variable all-fiber graphene device. (**b**) Gate-controlled Optical transition properties of the device. (**c**) Gate-controlled Electrical transport properties of the device. (**d**) Fiber laser configuration, including fabricated all-fiber device with bilayer graphene. LD: laser diode; EDF: erbium-doped fiber; PC: polarization controller; hybrid component: an integrated wavelength-division multiplexer and isolator. (**e**–**g**) Characteristics of a passively mode-locked fiber laser at an applied V_G_ of −1.05 V; (**e**) Measured pulse duration of 423 fs at a repetition rate of 30.9 MHz (inset). (**f**) Laser output spectrum with a spectral bandwidth of 8 nm at 3 dB. (**g**) The measured radio frequency spectrum of the laser output (**h**,**i**) Q-switched characteristics of a fiber laser at an applied V_G_ of −0.18 V; (**h**) Measured output pulse duration of 3.5 ms at a repetition rate of 25.4 kHz (inset) and (**i**) its optical spectrum. Reproduced with permission. Copyright 2015, Springer Nature [25].

Lee et al., for the first time, demonstrated electrically controllable all-fiber graphene SA operating in Q-switching and mode-locking regime depending on the external gate-bias [25]. CVD grown, high-quality large-area graphene capacitor structure was fabricated on the SPF surface using a droplet of ion liquid on it as shown in Figure 10a. When the gate voltage is applied, the ions form an electric double layer (EDL) at the liquid/graphene interface with effective capacitance thickness around 1 nm over the entire graphene area. The exceptionally elevated capacitance of the electric double layer directs to an effective change in the Fermi level of graphene, which noticeably tunes the optical transmission of the incident light on the graphene SA at comparably lower operation voltage. As shown in Figure 10b, at zero gate bias voltage (V_G_), the TE mode undergoes large absorption of 3.47 dB (>50%) (solid orange line at V_G_ = 0 V), while TM mode transmission rises by 0.09 dB (solid blue line at V_G_ = 0 V). This result is due to the reduced scattering loss caused by incorporating ion-liquid over-cladding on top of graphene and the polished surface.

More importantly, the applied gate voltage actively tuned the optical transmission of the device. TE mode exhibited a comparably larger change in transmission from 39.2 to 83.4% compared to the TM mode (from 87.1 to 90.8%) for V_G_ varying from 0.7 V to −1.8 V. This optical measurement implies that 44.2% of total incident light TE mode recovered after interacting with the single-layer graphene. These changes in optical transmission occur because of the Fermi level shift in graphene by an external gate voltage. Upon applying gate bias, the Fermi level near the Dirac point experiences substantial change due to incomplete density of state of electrons enclosed in a 2D graphene, revealing large electro-optic absorption. The corresponding change of electron carrier density in graphene was analyzed by determining the electrical transport properties of the device, as shown in Figure 10c. While the Fermi energy level reaches half the incident photon energy (*ħω/*2), the maximum transition in optical transmission appears to happen. The Fermi level change of 0.40 eV, which is the half-photon energy of the incident light source used in their experiment (1550 nm), is directly connected to the gate voltage deviation of 1.65 V, as described in Figure 10c. These integrated optical and electrical measurements and investigation could be a new technique to evaluate critical parameters like quantum capacitance and gate coupling efficiency without using complicated and expensive measurement systems. Nonlinear absorption characteristics of graphene such as saturation fluence and nonlinear modulation depth can be continuously tuned with the gate voltage, which can overcome the discrete nature of nonlinear absorption in stacked graphene sheets and be employed as functionally switchable devices. They also measured and observed the tunable nonlinear optical transmission as well as the varied modulation depth depending on the applied gate bias.

The fiber laser was built up using this device with a bilayer graphene SA case, which shows a self-starting of passive mode-locking operation at the applied V_G_ of −1.05 V where the measured pulse duration was 423 fs at a repetition rate of 30.9 MHz, as shown in Figure 10d–f. The spectral bandwidth of the laser output was measured to be 8.0 nm at the central wavelength of 1609 nm. The background noise level was >80 dB from the signal of the fundamental repetition rate of the laser output as shown in Figure 10g, which indicates stable mode-locking operation on increasing V_G_, the linear optical transmission decreases, while both modulation depth and saturation fluence of the SA increase. This significantly modifies the Q-switching instability condition, changing the fiber laser operation to Q-switching. Figure 10h,i shows measured Q-switched pulse duration (3.5 μs) and optical spectrum, respectively, at the applied V_G_ of −0.18 V. The repetition rate of the laser was measured as 25.4 kHz (inset of Figure 10h). For an applied voltage larger than 0.14 V or less than −1.65 V, the fiber laser turns to continuous-wave operation.

**Figure 11:**
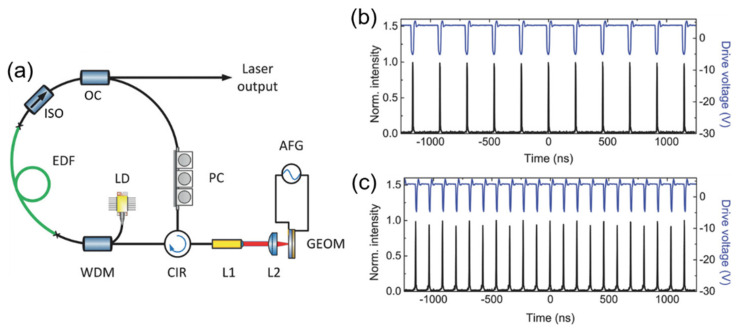
Graphene electro-optic modulator (GEOM) controlled mode-locked ultrafast fiber laser. (**a**) Laser cavity setup. EDF: erbium doped fiber; LD: laser diode; WDM: wavelength-division multiplexer; ISO: isolator; OC: output coupler; PC: polarization controller; CIR: circulator; L1: collimating lens; L2: focusing lens; AFG: arbitrary function generator. (**b**) Drive signal at the modulation frequency of 4.35 MHz and synchronized output optical pulse train for fundamental mode-locking operation at the repetition rate of 4.35 MHz. (**c**) Drive signal at modulation frequency of 8.7 MHz and synchronized output optical pulse train for second harmonic mode-locking operation at the repetition rate of 8.7 MHz. Reproduced with permission. Copyright 2018, Wiley-VCH [26].

Bogusławski et al. demonstrated an active mode-locked laser that achieved controlled ultrafast mode-locked laser pulses with tunable repetition rate by using a graphene-based electro-optic modulator (GEOM) as seen in Figure 11 [26]. The active mode-locking and active harmonic mode-locking of the erbium-doped fiber laser with output pulse duration of 1.44 ps and pulse energy of 844 pJ were achieved by the combination of the active mode-locking technique and the intracavity nonlinear pulse compression effect. The GEOM was integrated with an EDF laser working at 1.56 µm wavelength for active mode-locking. The experimental setup of the laser is presented in Figure 11a. The GEOM was inserted in a linear part of the cavity coupled with the ring-shaped part by a fiber circulator. A fiber collimator (L1) and an aspheric focusing lens (f = ≈4.6 mm, L2) were used to extract the beam outside the fiber and focus on the surface of the GEOM. The active mode-locking operation occurred immediately when the modulating signal frequency was set to precisely match the roundtrip frequency of the laser cavity (*f*_0_ = 4.3505725 MHz) and the pump power exceeds the CW lasing threshold of 18 mW. The modulating electrical signal with the amplitude and frequency of 8 V (from −4 to 4 V) and 4.35 MHz respectively, was applied to GEOM as drive voltage to see the fundamental mode-locking operation with the repetition rate of 4.35 MHz as shown in the pulse train (Figure 11b). As soon as the modulation signal frequency was raised to 8.7012 MHz, which corresponds to second harmonic of the laser cavity, the second harmonic mode-locking (HML) operation occurred at 1559 nm with the pulse repetition rate of 8.7 MHz as shown in Figure 11c.

**Figure 12:**
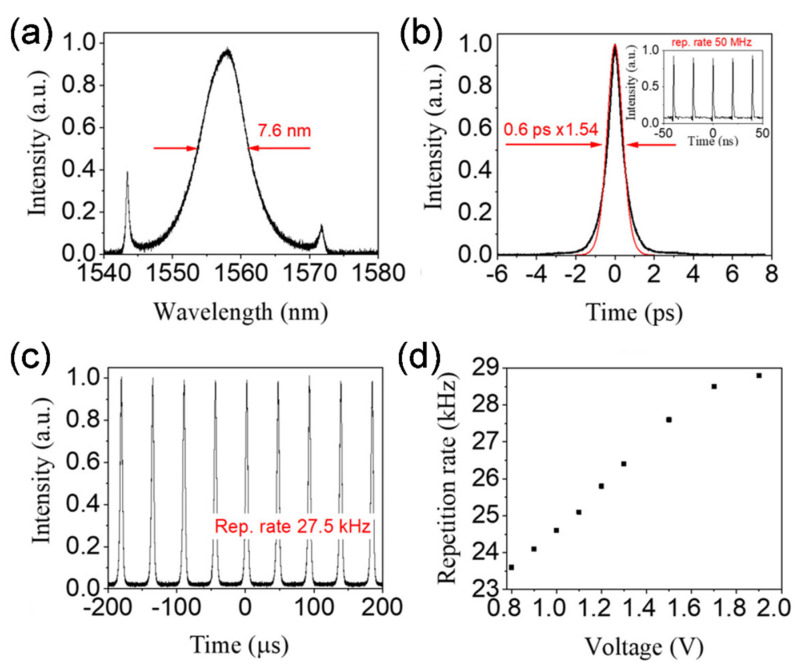
Electrically controlled fiber laser using SWCNT SA device and gate-variable properties of fiber laser operation. Soliton ML fiber laser characteristics at V_G_ = 0 V: (**a**) optical soliton pulse spectrum shows the 3 dB bandwidth of 7.6 nm, (**b**) autocorrelation trace shows the pulse duration of 600 fs with oscillation trace (inset showing repetition rate of 50 MHz) of the mode-locked pulse. QS fiber laser characteristics at V_G_ > 0.7 V: (**c**) oscillation trace of QS pulse with repetition rate of 27.5 kHz, (**d**) variation of QS pulse frequency as a function of applied gate voltage showing that, repetition rate is controlled in the range from 23.6 kHz to 28.8 kHz with applied gate voltage V_G_ varying from 0.8 V to 1.9 V. Reproduced with permission. Copyright 2019, American Chemical Society [67].

Gladish et al., in another work, reported that electrochemical doping could tailor the nonlinear optical absorption of SWCNT films and demonstrated its application to control pulsed fiber laser generation in a similar manner [67]. The SWCNTs were manufactured by the aerosol CVD technique and followed by a dry transfer technique to a polarization-maintaining side-polished fiber (PM-SPF) to execution of the fiber laser system. They exhibited a comprehensive mode-locked ultrafast pulsed laser employing this device capable of operating in both mode-locked and Q-switched regimes manipulated by the external gate voltage. Self-starting of mode-locking happened as the pump power was boosted up to 40 mW. Stable mode-locked lasing at the fundamental pulse repetition rate of 50 MHz and the spectrum width at half-maximum is 7.6 nm with 0.6 ps pulse duration was obtained at zero gate voltage as shown in Figure 12a,b. If the applied gate voltage reached over the threshold voltage of 0.7 V, the ML pulse disappeared, and the cavity operation regime was switched to the Q-switched laser. Microsecond pulses generated in the QS regime exhibited repetition rates in kHz regime (Figure 12c). If the gate voltage was further increased up to 1.9 V, the Q-switched laser was persistent with an increasing repetition rate. The repetition rate of the Q-switched laser pulse was tuned from 23.6 to 28.8 kHz by increasing the applied gate voltage from 0.8 V to 1.9 V (Figure 12d). The pulse energy of the laser was recorded as high as 12.5 nJ.

### 5.2. Optically Controlled Cross-Absorption Modulated Tunable Fiber Laser

**Figure 13:**
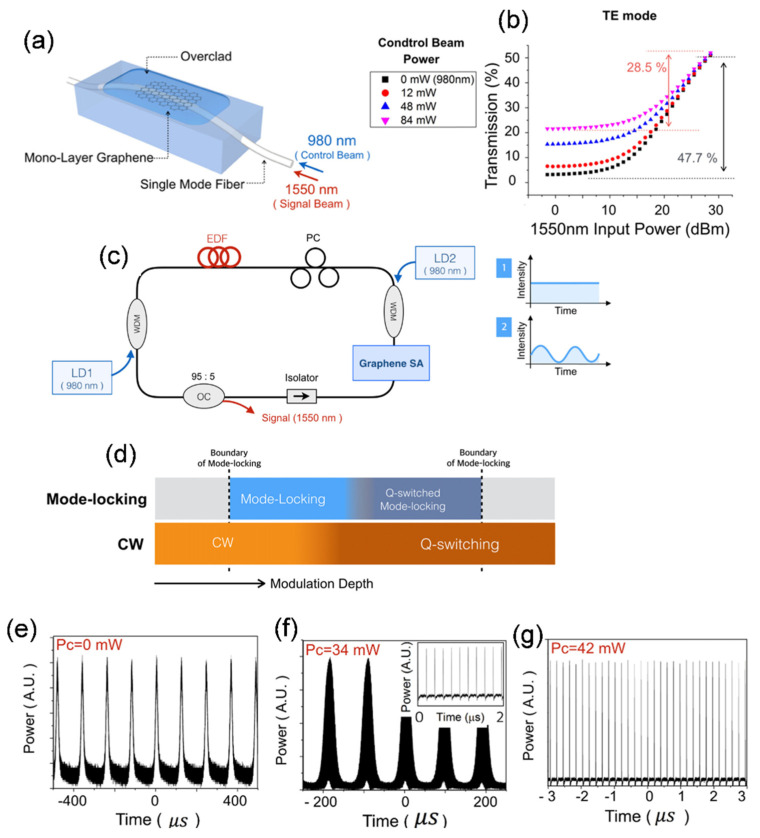
Optically controlled in-line graphene SA-based pulsed fiber laser (**a**) Schematic representation of optically tunable graphene SA. (**b**) Nonlinear transmission test result of the CW signal beam (1550 nm) TE mode variable with CW control beam powers at 980 nm. (**c**) Illustration of Er-doped fiber ring laser incorporated with optically controllable in-line monolayer graphene SA device. LD: laser diode; EDF: erbium-doped fiber; PC: polarization controller; WDM: wavelength-division multiplexer and OC: optical coupler. (**d**) Schematic explanation of fiber laser operating regime as a function of modulation depth in graphene SA, (**e**) Q-switching operated pulse train with no control beam (P_c_ = 0 mW) applied, (**f**) Q-switched mode-locked operated pulse train with a control beam power of 34 mW (inset: an extended view of the pulse train in time scale) and (**g**) pulse train of CW mode-locked operation with a control beam power of 42 mW. Reproduced with permission. Copyright 2016, OSA [68].

Apart from the electrical control, optically excited carriers also alternatively tune the linear optical absorption in graphene through Pauli blocking theory, which facilitated graphene-based devices for all-optical modulation in broadband scale [206]. This can adjust the optical properties of graphene SA on a nonlinear scale, such as modulation depth and non-saturable loss. This method was utilized in the fiber laser cavity by Sheng et al. to optically manipulate and tune the pulse width of a passively mode-locked ultrafast fiber laser based on graphene SA [207]. Cross absorption modulation (XAM) employing evanescent field interaction significantly modified the nonlinear SA in graphene. Gene et al., in the recent past, successfully accomplished different pulsed fiber laser regimes by optically controlling and manipulating the SA [68]. They displayed optically tuned in-line graphene SA with higher tunability in modulation depth by means of boosting the interaction of graphene SA with the incident evanescent field. The all-fiber graphene SA was manufactured by transferring a uniform and large-area single-layer graphene onto the side-polished fiber (SPF), as seen in Figure 13a. Nonlinear absorption properties of graphene SA were optically controlled by means of XAM in a graphene sheet. A shorter wavelength (980 nm) light was utilized to manipulate the absorption of the signal beam at a higher wavelength (1550 nm). As the graphene SA layer absorbed the control beam, the signal beam experienced less absorption by XAM [207]. In the case of the parallel polarization direction of TE mode along with the graphene layer at a low power level, the incident signal beam encounters only a slight transmission of 2.5% in the monolayer graphene as shown in Figure 13b. At a high-power level, 50.2% of the transmission recorded in the absence of the applied control beam reveals the modulation depth of 47.7% over the 800 mW of signal power. The modulation depth reduced to 28.5% as soon as the incident control beam power reached 84 mW. The graphene SA device was incorporated into an Er-doped fiber ring-laser system, as illustrated in Figure 13c. The laser cavity comprises two wavelength division multiplexing (WDM) couplers and two 980-nm laser diodes (LDs), pumping the EDF and the other to control the graphene SA. A pulsed fiber laser is capable of operating in several modes such as Q-switching, Q-switched mode-locking (QML), and CW mode-locking mode, which can be governed by manipulating the system parameters such as modulation depth, gain relaxation time, gain saturation power, small-signal gain, and saturation power of the SA. Figure 13d shows the switching performance of the fiber laser regime as a function of SA modulation depth. Figure 13e–g illustrates experimental findings of the switching actions altered between different laser operating regimes achieved through active control graphene SA by manipulating the external control beam in a pulsed fiber laser setup. In the absence of a control beam (P_c_ = 0 mW), graphene SA in the ring cavity reveals the Q-switched laser (Figure 13e), with the pulse duration and repetition rate of 20μs and 8.0 kHz, respectively. The laser state was switched to QML if the control beam power was elevated to 34 mW. A fine mode-locked pulse train at a repetition rate of 5.09 MHz (inset of Figure 13f) is clearly observed within the 20 kHz Q-switched pulses, as shown in Figure 13f. CW mode-locking status was achieved as the control beam power was increased to 42 mW (Figure 13g). In addition to these, they also examined the laser characteristics when a control beam with sinusoidal modulation frequency was applied to the graphene SA at a particular power of the control beam. It was seen that the generated Q-switched laser emitted from the cavity exhibited the repetition rate same as the modulation speed of the applied control beam. A Q-switched laser with a continuously tunable repetition rate in the range from 6.2 kHz to 11.8 kHz was obtained by manipulating the modulation frequency of the control beam. The controllable and switchable fiber pulse laser point towards a suitable solution for developing versatile pulse laser seed sources. This kind of source can potentially find a number of applications both in Q-switching and mode-locking regimes (for example, remote sensing, distance measurement, time-resolved spectroscopy, optical frequency metrology, or photo-acoustic imaging). This concept can be further expanded to an optical fiber taper that potentially provides more tight confinement of light, resulting in reduced device size with the higher operating speed of the device. The proposed scheme can also be applied to the plastic optical waveguides combined with an ionic gel, paving a novel way for actively controlled, flexible photonic devices. Based on this and combined with the fundamental studies, high repetition rate ultrafast fiber lasers or tunable repetition rate Q-switched fiber lasers also can be considered for future works. These investigations of graphene and CNT for externally controlled fiber lasers are also exciting to explore other LD SAs, especially 2D SAs, to find efficient optical devices with high performance and precisely tunable functionalities.

## 6. Prospects for Future Research Directions

Low-dimensional materials, particularly 2D materials, are a rising and hot topic expanding in quality, variety, and quantity. Over the last decade, the development of pulsed lasers based on LD materials has progressed rapidly, yielding many significant results and being employed in various applications. This is due to the reliability of synthesis of LD materials, coupling technology to implement SA devices, and the steady advancement of pulsed fiber laser technology over the decades. Nevertheless, there are still numerous challenges to realize high performance and finely tunable pulsed fiber laser and technical as well as scientific issues to be solved. Controllability of fiber laser in between Q-switching and mode-locking has been achieved by electrically and/or optically tuning the Fermi level, the modulation depth, and cross absorption modulation of LD materials. However, in the case of ion-liquid over-cladding, the switching speed and stability are limited by the ionic mobility in the ion liquid. This prospects towards the development of stable over-cladding in the capacitor structure based on LD SA on SPF that will benefit the stability and specifically control the mode-locking regime. Numerous stable over-cladding indexes and solid polymer indexes have been developed to study and analyze the LD SA-based mode-locked fiber laser and photonics characteristics. However, the externally controlled or gate-tunable LD SA-based fiber laser has not been studied yet, employing the stable over-cladding index required for enhanced coupling as well as for the EDL formation, which is crucial for the Fermi level tuning in the LD SAs. Moreover, only 2D graphene and 1D CNT among a large variety of LD SAs has been investigated for a tunable mode-locked fiber laser. There are many other LD SAs to be analyzed in this specific field for a stable, high-performance controllable fiber laser. Additionally, the basic characteristics of the ultrafast fiber laser, such as repetition rate and pulse duration, may be effectively controlled utilizing the tunable behavior of LD SA upon external (electrical and optical) bias. All of these LD-based tunable devices could also be investigated for various in-line all-fiber devices, such as ultrafast all-optical tunable switchers, optical limiters, all-optical modulators, polarizers etc., based on electrically/optically controlled nonlinear optical properties (i.e., higher-order susceptibility/nonlinearity, multiphoton absorption, etc.). The twist-angle in Bi-layer LD materials also can be engineered along with the externally (electrically and/or optically) controlled scheme to find the enhanced higher-order optical nonlinearity and SA properties for high performance, ultrafast and precisely controlled fiber lasers, and other nonlinear optical devices [54]. LD SA-based fs mode-locked ultrafast fiber lasers have grown emerging interest in space-borne applications, as they can sustain more than the life span of satellites [136,208,209]. Other radiation environments such as particle accelerators and radiation-based medical instruments also could be benefit from utilizing the controllable functionality of ultrafast fiber lasers based on actively controlled characteristics of LD SAs. The tunability of such fiber lasers could provide significant functionality in that application for the development of future space technology and many other advanced applications. 

## 7. Conclusions

In conclusion, we present a brief review of numerous LD SA-based ultrafast fiber lasers and their performance reported so far in various technical schemes. Based on the aforementioned review, we estimate that LD materials with broadband optical response, high stability, good reliability, excellent thermal performance, low defects, and precisely controllable properties, which are compatible for high-energy ultrafast fiber laser along with the functional operation, will be constructed and taken out of the laboratory for real-world applications. Although there have been several reports on the electrically controlled nonlinear transmission and saturable absorption in graphene and carbon nanotube-based mode-locked ultrafast fiber laser and Q-switched laser, there is still more room to explore externally controlled saturable absorbers based on other LD SAs. The electrical and optical gating of graphene and SWCNT exposes the opportunities for the development of externally (electrically or optically) tunable other various LD-materials based nonlinear optical devices and points towards advanced device performances with tunable nonlinearity and controllable functionalities. Principally in both practical and theoretical frameworks, the externally (electrically and/or optically) tunable nonlinear optical operation of 2D graphene and other LD nonlinear optical materials also propose a variety of technical and scientific benefits, such as devices with a compact minimum footprint, exceedingly fast speed (more than a few tens of GHz), chip-scale integration and compatibility with complementary metal-oxide-semiconductor (CMOS) technology, all of which are required ideal standards for future on-chip photonic and optoelectronic applications. These will also benefit the basic study and investigation of LD materials for further understanding the incomparable advantages of nonlinear optical as well as ultrafast photonic systems over their electronic counterparts.

## Figures and Tables

**Table 2:** Performance summary of Externally controlled LD SA for tunable fiber laser.

LD SA (Type of Control)	Operating Voltage (V) or Control Beam Power (mW)	Fiber Laser Regime (QS or ML)	Repetition Rate (MHz)	Pulse Duration (ps)	3 dB Bandwidth (nm)	Center Wavelength (nm)	Ref.
Bi-layer graphene (Electrically controlled-ion-liquid gated)	−1.05 V	ML	30.9	0.423	8	1609	[25]
	−0.18 V	QS	25.4 kHz	3.5 μs	-	1590	
Bi-layer Graphene(Electrically controlled-PMMA)	±4 V	ML	2.44	0.390	8.9	1547.5	[66]
	-	QS-Not tested	-	-	-	-	
Mono-layer Graphene (Electro-optic modulator)	8 V (−4 to +4 V, 4.35 MHz)	ML	4.35	1.44	1.8	1559.2	[26]
	8 V (−4 to +4 V, 8.70 MHz)	HML	8.70	1.57	1.82	1559.3	
Bi-layer Graphene (Electro-optic modulator)	3.5 V (35 to 65 KHz)	QS	35 to 65 KHz	1.93 to 5.54 μs	0.05	1524.6 to 1561.7	[27]
SWCNT (Electrically controlled-ion-liquid gated)	0~0.7 V	ML	50	0.6	7.6	1558	[67]
	0.8~1.9 V	QS	23.6~28.8 kHz	-	-	1559	
Monolayer graphene (Optically controlled- ion-liquid gated), Biased by 980 nm CW pump beam	42 mW (980 nm)	ML	5.09	0.980	2.6	1570	[68]
	34 mW (980 nm)	QS-ML	20 kHz 5.09 MHz	-	-	-	
	0 mW (980 nm)	QS	8 kHz	20 μs	-	-	
	30 mW Modulated signals	QS	6.2~11.8 kHz	-	-	-	
	Square pulse (1 ms)	QS	1 Hz	-	-	-	

## Data Availability

Data is contained within the article which are from various articles cited as references.
